# The Anti-Apoptotic Role of Neurotensin

**DOI:** 10.3390/cells2010124

**Published:** 2013-03-04

**Authors:** Christelle Devader, Sophie Béraud-Dufour, Thierry Coppola, Jean Mazella

**Affiliations:** Institut de Pharmacologie Moléculaire et Cellulaire, CNRS UMR 7275, Université de Nice-Sophia Antipolis, 660 route des Lucioles, Valbonne 06560, France; E-Mails: devader@ipmc.cnrs.fr (C.D.); beraud@ipmc.cnrs.fr (S.B.-D.); coppola@ipmc.cnrs.fr (T.C.)

**Keywords:** neurotensin, receptor, apoptosis, sortilin

## Abstract

The neuropeptide, neurotensin, exerts numerous biological functions, including an efficient anti-apoptotic role, both in the central nervous system and in the periphery. This review summarizes studies that clearly evidenced the protective effect of neurotensin through its three known receptors. The pivotal involvement of the neurotensin receptor-3, also called sortilin, in the molecular mechanisms of the anti-apoptotic action of neurotensin has been analyzed in neuronal cell death, in cancer cell growth and in pancreatic beta cell protection. The relationships between the anti-apoptotic role of neurotensin and important physiological and pathological contexts are discussed in this review.

## 1. Introduction

The tridecapeptide neurotensin (NT) was isolated from bovine hypothalami on the basis of its ability to induce vasodilatation [1]. NT is synthesized from a precursor protein following excision by prohormone convertases [2]. Since NT is expressed both in the central nervous system and in the gastrointestinal tract [3], the peptide exerts a role of a neurotransmitter/neuromodulator in the central nervous system and a role of a hormone in the periphery.

In the central nervous system, NT is released from neuronal synaptic vesicles [4]. The peptide regulates the dopaminergic system [5], induces an opioid-independent analgesia [6] and a potent hypothermia [7]. A role for NT in schizophrenia was suggested in 1980 [8] and further confirmed by the observation that NT level decreases in the brain of severe schizophrenics [9]. Low NT level can be reversed to a normal level after one month treatment with anti-psychotic drugs [10].

In the periphery, NT is released in the circulation after a meal [11] and stimulates pancreatic secretion in order to facilitate lipid digestion [12]. NT is also involved in the protection of pancreatic beta cells against cytotoxic molecules [13] and in the regulation of insulin secretion [14,15].

The numerous activities of NT are mediated through three identified NT receptors (NTSRs). Two of them, NTSR1 and NTSR2, are classical neuropeptide G protein coupled receptors (GPCR) bearing seven transmembrane domains (TMs) [16,17]. The third one, NTSR3, also called sortilin [18,19], is a type I receptor, non-coupled to G proteins, which belongs to the Vps10p-containing domain receptor family [20,21].

The homeostasis of tissues results from a fine equilibrium between cell differentiation, proliferation and apoptosis. Both proliferation and apoptosis display crucial roles in the growth control and development of normal and neoplastic tissues. Therefore, dysregulation of one of the pathways controlling proliferation or apoptosis leads to carcinogenesis by dysregulation of normal cell cycle [22]. Apoptosis is regulated by multiple intracellular pro- and anti-apoptotic proteins. 

The protein B-cell lymphoma/leukemia 2 (Bcl-2) was the first mitochondrial protein shown to block apoptotic programmed cell death [23,24]. Since the discovery of Bcl-2, a number of proteins sharing structural homology and involved in the control of apoptosis has been characterized [25]. Members of the Bcl-2 family are globular proteins structured in α-helices, which possess at least one Bcl-2 homology (BH) domain. However, not all Bcl-2 family members have the same anti-apoptotic function. Bcl-2, Bcl-xL and Mcl-1 are anti-apoptotic; they contain four BH domains and a transmembrane (TM) domain. The proteins Bax and Bak, which also contain four BH domains and a TM domain, are pro-apoptotic. A second group of pro-apoptotic proteins possessing only one BH domain is composed of Bik and Hrk (with a TM domain) and Bad and Bid (without TM domain) [26,27]. Bcl-2, like other anti-apoptotic members, is able to form heterodimers with pro-apoptotic proteins, like Bax. This prevents the permeabilization of the outer mitochondrial membrane and then the release into the cytosol of cytochrome C, responsible for the activation of the caspases cascade [28]. Following cellular stresses, the p53 tumor suppressor, which is another representative of pro-apoptotic proteins, limits cell growth by inducing cell cycle arrest and apoptosis. Since p53 mediates apoptosis through a pathway involving Bax, its activity can be blocked by anti-apoptotic Bcl-2 family members [29]. Numerous studies demonstrated a role of NT in the regulation of apoptosis through the activation of the Bcl-2 family proteins. In the present review, we will focus on the history of the discovery of the anti- and, with a lesser extent, the pro-apoptotic action of NT both in central and peripheral tissues.

## 2. Anti-Apoptotic Actions of NT in the Central Nervous System

The neurotensinergic system is altered in disease-related neurodegeneration. In the temporal lobe of Alzheimer’s disease (AD) patients, the expression levels of both NTSR1 and NTSR2 were profoundly decreased in AD, whereas the expression of NT was slightly reduced, and the level of NTSR3/sortilin did not vary [30]. NT and its receptors are also modulated during age-related neurodegeneration. A study demonstrated that the absence of NTSR3/sortilin prevents age-related neuron degeneration, but not developmental neuronal apoptosis [31]. Also, in the retina, NTSR3/sortilin-deficient mice showed reduced neuronal apoptosis [31]. Although the neurotensinergic system is clearly modified during ageing and neurodegenerative diseases like AD, its functional involvement was not established.

However, it is important to remember that NTSR3/sortilin is able to form heterodimers with neurotrophins receptors, like p75NTR or TrkA and TrkB, to trigger proNGF or proBDNF-induced neuronal cell death (for a review, see [32]). The first demonstration of the involvement of NTSR3/sortilin in neuronal cell death was performed in 2004 in an original work showing that both sortilin and p75NTR form a protein complex, which is crucial for the apoptotic effect of the precursor form of NGF (proNGF) on neurons [33]. Interestingly, this work provides also evidence for a protective role of NT on neurons. The peptide antagonizes the effect of proNGF by competition on the binding site of proNGF on sortilin [33]. Moreover, another work demonstrated that in ageing rodent basal forebrain and sympathetic neurons, both proNGF and sortilin expression were increased, whereas p75NTR levels remained unchanged [34]. In this context, sortilin was involved in the age-related proNGF-mediated neurotoxicity, and the survival of aged neurons was rescued by NT.

Further works have shown that proBDNF-induced neuronal apoptosis involves a receptor complex formed also by sortilin and p75NTR. In this system, NT is also able to counteract the proneurotrophin-induced apoptosis by competition for the sortilin binding site [35]. Another neurotrophin precursor (proNT3) uses the same protein complex (sortilin/p75NTR) to trigger inner ear neuron death [36]. In this case, the role of NT has not been investigated.

Sortilin has been shown to participate in light-dependent photoreceptor degeneration *in vivo*. This work evidenced that the expression of p75NTR, sortilin and proNGF is increased following intense illumination, a process leading to retinal cell death, likely through proNGF-induced neurodegeneration. Pharmacological blockade of sortilin with either NT (10 µM) or the pro-domain of proNGF (100 nM) enhances the survival of cone-progenitor 661W cells subjected to intense light. This demonstrated that NT and the pro-domain of proNGF are able to antagonize, by competition to the binding site of sortilin, the proNGF-induced cell apoptosis [37]. However, the concentration of NT used in this work is largely higher than the physiological concentration of the peptide, which varies from 50 pM in the brain [38] to 100 pM in plasma serum [39]. Taking into account that NT is expressed in amacrine cells of the retina [40] and the three known NT receptors have been detected in human corneal keratocytes [41], NT could serve as a physiological regulator. NT can protect from intense illumination damage, as suggested by the action of the NT analogue JMV449, which at a physiological concentration of 1 or 10 nM decreases keratocyte apoptosis [41].

Finally, an important protective effect of NT through its interaction with the NTSR1 has been demonstrated in a stroke model of focal cerebral ischemia [42]. In this work, the NTSR1 agonist ABS-201, a stable NT analogue originally developed for its ability to cross the blood-brain barrier [43], protected adult mice brain from focal ischemia by decreasing body and brain temperature. ABS-201 treatment immediately or 1h after the ischemia leads to a significant decrease (40%) of infarct volumes. The interaction of ABS-201 with NTSR1 decreases ischemia-dependent caspase-3 activity and increases Bcl-2 expression [42]. These results are crucial, since pharmacological induced hypothermia elicited by NT analogues represented by ABS-201 are promising candidates for treatment of ischemic stroke and possibly for other ischemic or traumatic injuries.

## 3. Anti-Apoptotic Effects of NT in Non-Neuronal Cell Types

### 3.1. Cancer Cells

NT is known to induce cell proliferation, both in normal and cancer tissues, and to promote neuronal protection [17]. However, the cellular mechanisms, apoptotic or survey pathways, which are activated to trigger these effects, remain undefined in several cases. NTSRs have been shown to be overexpressed in tumors and preferentially in cancers from pancreatic, prostatic and mammary origins [44]. The first demonstration that NT modulates the apoptotic pathway was performed on the breast cancer cells, MCF-7 [45]. In this work, long exposure (96 h) of the stable NT agonist JMV449 inhibits apoptosis and stimulates cell proliferation in MCF-7 cells. This action is mediated by an increase of Bcl-2 mRNAs and protein expression. However, the increase of Bcl-2 is not followed by a decrease of p53 and caspase-3 expression, suggesting that apoptosis observed in resting cells is independent of p53. The increase of proliferation and the decrease of apoptosis mediated by the NT analogue JMV449 have been also observed in human corneal keratocytes. Both NTSR1 and NTSR3 are expressed in this tissue; however, the cellular pathway leading to these effects has not been investigated [41]. Several studies indicate that the effect of NT on cancer cells is mediated by NTSR1. The selective NTSR1 antagonist SR48692 increases the efficiency of ionizing radiation for treatment of prostate cancers [46]. Moreover, NTSR1 inhibition leads to the attenuation of epidermal growth factor receptor activation and downstream signaling [44]. Finally, SR48692 efficiently radiosensitized PC-3M orthotopic human tumor xenografts in mice and significantly reduced tumor burden [46]. These findings offer preclinical proof of concept for targeting the NTSR1 receptor as a strategy to improve efficacy and outcomes of prostate cancer treatments using radiotherapy.

Interestingly, targeting the ligand instead of the receptor may also have an impact on cancer growth. The Neutral Endopeptidase (NEP or E-24-11) efficiently suppresses tumor progression by its pro-apoptotic action [47]. The effects of NEP are mediated by its ability to catalytically inactivate substrates, such as bombesin [48], endothelin-1 [49] or NT [50]. NEP reduces the local concentration of peptide available for receptor binding and signal transduction. Thus, NEP may decrease the proliferative effects of peptides, such as NT, that are involved in cancer progression.

### 3.2. Gastrointestinal Tissues and Cells

A series of studies performed on liver and on the gastrointestinal tract revealed a marked protective action of NT. Peptide administration significantly reduces portal and systemic endotoxemia observed in obstructive jaundice. NT reverses obstructive jaundice-induced morphologic features of intestinal atrophy by increasing villus density and mucosal thickness. These effects were accompanied by a reduction of apoptosis in intestinal crypts [51]. This observation might be of potential value in patients with extrahepatic cholestasis. Investigation of the action of NT on intestinal barrier function in partially hepatectomized rats demonstrated a proliferative effect of the peptide. This trophic effect of NT is generated by induction of mitoses above control levels and also by a significant reduction of apoptosis in intestinal crypts [52]. Thus, NT exerts a proliferative effect on hepatocytes by inhibiting apoptosis, as measured by terminal deoxynucleotidyl transferase dUTP nick end labeling (TUNEL). However, the peptide reduces the proliferation of oval cells (hepatic stem cells activated after injury) with an increase of the apoptotic index of cholangiocytes [53]. In the colitis model induced by trinitrobenzene sulfonic acid injection in the rat, a significant increase in the levels of proinflammatory cytokines and apoptosis was observed. In this model, NT reduces the levels of the proinflammatory cytokines interleukin-6 (IL-6) and tumor necrosis factor-α (TNF-α); as a consequence, NT decreases inflammation and the rate of apoptotic death, as measured by caspase-3 activity [54].

### 3.3. Pancreatic Tissues and Cells

Until recently, NT was mostly known to regulate insulin secretion from beta cell lines and islets. Indeed, at low glucose concentration, NT stimulates insulin release, whereas at high glucose level, NT decreases the glucose-induced insulin secretion [14]. Recently, NT was shown to be involved in the protection of both pancreatic beta cells and of islets from cytotoxic agents, like Il-1β and staurosporine [13]. This protective effect is characterized by a strong decrease (40%) of Il-1β and staurosporine-induced caspase-3 activity. The protection of pancreatic secreting cells is physiologically relevant, since NT is released in the circulation following a meal and particularly after fat ingestion, which is cytoxic for beta cells. Interestingly, the protective effect of NT through Akt activation on beta cells is dependent on a heterodimeric protein complex formed between NTSR2 and NTSR3 [55].

### 3.4. Immune System

As previously demonstrated in the central nervous system, NTSR3/sortilin has been shown to form a functional complex with the NGF receptor p75NTR in cells of the immune system. Indeed, in B-cells, BDNF is involved in cell survival, likely through its interaction with its tropomyosin-related kinase B (TrkB) receptor [56]. Serum starvation of B-cells increases the ratio of apoptotic cells, likely through the release of ProBDNF and its interaction with the complex p75NTR/sortilin. In these conditions, NT, by its ability to bind sortilin, exerts a protective effect on B-cells against apoptosis. Interestingly, both NTSR1 and NTSR2 are expressed in normal and malignant human B-cell lines [57]. In these cell lines, NT acts directly through its own receptors to induce proliferation and to inhibit cell apoptosis activated by serum deprivation or by ligands of death receptors (Fas ligands) [57].

More recently, under exposition to interleukin-12 (IL-12), p75NTR, NTSR3/sortilin and proNGF were found to be expressed in Natural Killer (NK) cells. As expected, proNGF induces apoptosis of NK cells leading to cell death through activation of the p75NTR-sortilin complex. This effect was efficiently reduced by blocking sortilin binding sites with NT [58].

Finally, in fetal-skin dendritic cells, NTSR1 and NTSR3 are constitutively expressed, and LPS treatment induces NT expression [59]. In this model, NT downregulates the inflammatory signaling pathways NF-κB and JNK and the expression of the cytokines IL-6, TNFα, IL-10 and the vascular endothelial growth factor (VEGF). Interestingly, NT upregulates the survival pathway ERK and epidermal growth factor (EGF). However, when cells are pre-treated with NT and then incubated with LPS, the cytokines tested are increased without change on growth factor expression. This work is of importance for new therapies using neuropeptides for skin diseases [60]. However, further studies are necessary in other skin cells and *in vivo* conditions, in order to confirm the potential of NT for treatment of skin pathologies.

## 4. Pro-Apoptotic Action of NT in the Central Nervous System

We cannot complete this review without describing some properties of NT that lead to a pro-apoptotic action of the peptide, essentially observed in the central nervous system. In the cultured cortical neurons model, glutamate exerts exocytotoxicity via *N*-methyl-D-aspartate (NMDA) receptors, leading to an apoptotic cell death [61]. It has been shown that NT significantly enhances glutamatergic signaling, both *in vitro* and *in vivo*, by increasing glutamate release [62,63,64,65]. The fact that the cerebral level of NT increases following ischemia [66] could suggest an involvement of the peptide in ischemic brain damage. However, other experiments provide evidence that NT-induced hypothermia improves neurologic outcome and reduces infarct volume after hypoxic ischemia [67]. Indeed, the neuroprotection caused by NT-induced hypothermia *in vivo* [42,68] is not in agreement with the enhanced pro-apoptotic action of glutamate by NT after oxygen and glucose deprivation of primary cortical cultures [69]. The latter authors hypothesized that the difference in the NT effects may be explained by the influence of temperature. Experiments carried out on primary cultures of neurons are performed *in vitro* under normothermic conditions, whereas, in experiments achieved *in vivo*, animals can modulate their temperature. As NT can decrease body and brain temperature, the cytotoxic effect of glutamate is likely partially or even totally blocked or reversed. Further experiments on cultured neurons carried out under reduced temperature, 34 °C instead of 37 °C to mimic NT-induced loss of temperature, should answer this problem.

## 5. Discussion

**Figure 1:**
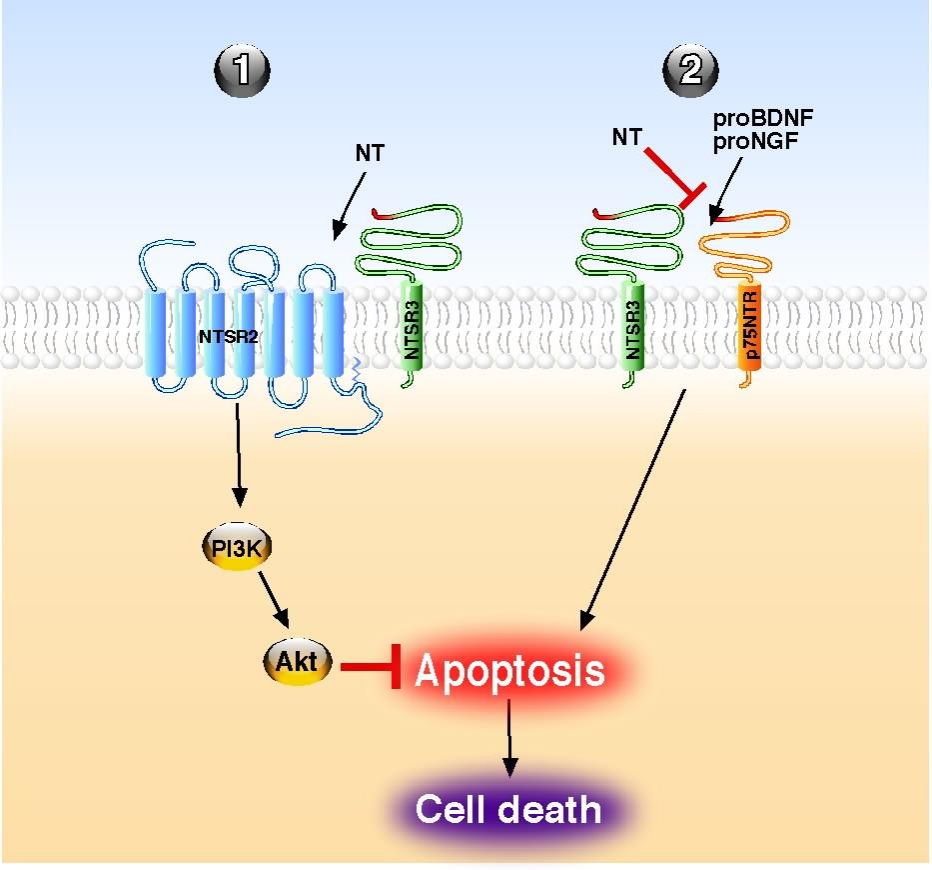
Molecular mechanisms of NT on apoptosis regulation. NT acts directly through its receptors to block the apoptosis (**1**) or indirectly by competition to reverse the proneurotrophin-induced apoptosis (**2**). PI3K; phosphatidyl inositol 3 kinase, NT; neurotensin.

Although NT has been also shown to stimulate the release of inflammatory cytokines and chemokines from peripheral cancer cells [70] and from central microglial cells [71], the overall action of the peptide, as referenced in this review, is a protective effect (Table 1). This cellular and tissue protection is essentially mediated by a broad spectrum of an anti-apoptotic action of the peptide both in the periphery and in the central nervous system. The role of NT against cell apoptosis is triggered either by its classical GPCR NTSR1 and NTSR2 or by the NTSR3/sortilin, a non-G protein coupled receptor. The NT GPCRs usually mediate their action either directly as demonstrated for the protection of brain infarct against ischemia [42] for NTSR1 or in combination with NTSR3/sortilin, as shown for the protective action of NT on pancreatic beta cells [55]. Inversely, the involvement of NTSR3/sortilin is always made within a protein complex containing other NT receptors or receptors of neurotrophins, like p75NTR. However, when NTSR3/sortilin is associated with another NT receptor, the anti-apoptotic effects of NT are direct through NTSRs activation, as exampled in beta cells [55], and in human colonic cancer cells [72] (Figure 1). By contrast, when NTSR3/sortilin is associated with p75NTR, NT itself does not trigger receptor complex activation, but the peptide antagonizes the pro-apoptotic effect of either proNGF [33] or proBDNF [35] by competition with the pro-domains for the same binding site (Figure 1).

**Table 1:** Summary of the central and peripheral effects of tridecapeptide neurotensin (NT) on the regulation of cell apoptosis (n.d.: not determined).

Site	Effect	Receptor(s)	Target	Cellular pathway	References
Brain	antagonism of pro-NGF-induced apoptosis	NTSR3/sortilin	neurons	n.d.	[33]
Brain	antagonism of pro-BDNF-induced apoptosis	NTSR3/sortilin	neurons	n.d.	[35]
Brain	reversion of ischemia-induced apoptosis	NTSR1	neurons	Bcl-2	[42]
Brain	increase of glutamate-induced apoptosis	n.d.	neurons	n.d.	[69]
Retina	inhibition of natural apoptosis of primary cultures	NTSR1	corneal keratocytes	n.d.	[41]
Retina	reversion of light-induced apoptosis	NTSR3/sortilin	retinal cells		[37]
Breast	inhibition of serum deprivation-induced apoptosis	NTSR1	MCF-7 cells	Bcl-2	[45]
Liver	inhibition of bile duct ligation-induced apoptosis	n.d.	oval cells	n.d.	[53]
Intestine	reduction of colitis-induced apoptosis	NTSR1	intestinal tissue	n.d.	[54]
Intestine	reversion of jaundice-induced intestinal atrophy	n.d.	intestinal crypts	n.d.	[51]
Endocrine pancreas	protection against Il-1β-induced apoptosis	NTSR2–NTSR3/sortilin	beta cells	Akt	[13,55]
Immune system	reversion of pro-BDNF-induced apoptosis	NTSR3/sortilin	B-cells	n.d.	[56]
Immune system	reversion of serum deprivation-induced apoptosis	NTSR1–NTSR2	B-cells	n.d.	[57]
Immune system	antagonism of pro-NGF-induced apoptosis	NTSR3/sortilin	NK cells	n.d.	[58]

## 6. Conclusions

In conclusion, on one hand, the anti-apoptotic action of NT is beneficial when it concerns the protection of cells or tissues against cytotoxic agents (IL-1β, proNGF, proBDNF). On the other hand, the anti-apoptotic effect of NT is deleterious when it concerns the growth of neoplastic cells. The latter case necessitates the blocking of NT receptors, which are overexpressed in tumors. Therefore, the use of NT or NT analogues in therapy remains difficult to set up without taking into account these particular properties.
